# Chelate Coordination Compounds as a New Class of High-Energy Materials: The Case of Nitro-Bis(Acetylacetonato) Complexes

**DOI:** 10.3390/molecules26185438

**Published:** 2021-09-07

**Authors:** Danijela S. Kretić, Ivana S. Veljković, Aleksandra B. Đunović, Dušan Ž. Veljković

**Affiliations:** 1 University of Belgrade-Faculty of Chemistry, Studentski trg 12-16, 11000 Belgrade, Serbia; danijela@chem.bg.ac.rs; 2 University of Belgrade-Institute of Chemistry, Technology and Metallurgy, Department of Chemistry, Njegoševa 12, 11000 Belgrade, Serbia; ivana.veljkovic@ihtm.bg.ac.rs; 3 Innovative Centre of the Faculty of Chemistry, Studentski trg 12-16, 11000 Belgrade, Serbia; aleksandra.djunovic@gmail.com

**Keywords:** high-energy materials, electrostatic potential, DFT calculations

## Abstract

The existence of areas of strongly positive electrostatic potential in the central regions of the molecular surface of high-energy molecules is a strong indicator that these compounds are very sensitive towards detonation. Development of high-energy compounds with reduced sensitivity towards detonation and high efficiency is hard to achieve since the energetic molecules with high performance are usually very sensitive. Here we used Density Functional Theory (DFT) calculations to study a series of bis(acetylacetonato) and nitro-bis(acetylacetonato) complexes and to elucidate their potential application as energy compounds with moderate sensitivities. We calculated electrostatic potential maps for these molecules and analyzed values of positive potential in the central portions of molecular surfaces in the context of their sensitivity towards detonation. Results of the analysis of the electrostatic potential demonstrated that nitro-bis(acetylacetonato) complexes of Cu and Zn have similar values of electrostatic potential in the central regions (25.25 and 25.06 kcal/mol, respectively) as conventional explosives like TNT (23.76 kcal/mol). Results of analysis of electrostatic potentials and bond dissociation energies for the C-NO_2_ bond indicate that nitro-bis(acetylacetonato) complexes could be used as potential energetic compounds with satisfactory sensitivity and performance.

## 1. Introduction

The design and preparation of new High-Energy Materials (HEM) with improved stability and satisfactory performance attracted extensive attention of scientists working in the field of energy compounds over the last few decades [1,2,3,4]. Many classical explosives fall into the categories of nitroaromatic or nitroaliphatic compounds, however, these energetic molecules usually have very high sensitivities towards detonation. Although nitroaromatic and nitroaliphatic compounds are the focus of the vast majority of studies in this area, coordination compounds are also seen as possible energy molecules with promising detonation characteristics [4,5,6,7,8,9,10,11].

The main challenge in the development of new HEM compounds is achieving the balance between high and low sensitivity towards detonation, since energy compounds with good performance are usually very sensitive towards mechanical stimuli [2]. Establishing control over the impact sensitivities of energetic molecules is very challenging because many electronic and crystalline factors affect the sensitivities of HEM molecules towards detonation. Some of the most important factors that affect HEM sensitivities towards detonation are energy content, oxygen balance, voids, and free space per molecule in the crystal lattice, hydrogen bonding, and positive values of electrostatic potential in the central regions of the molecular surface [3,12,13,14,15].

The positive value of molecular electrostatic potential (MEP) above the central regions of the molecular surface is recognized as an especially important indicator of the high sensitivity of many energetic molecules [2,16]. Positive electrostatic potential induces resistance to the shifting and slipping of the lattice planes in the crystal structures of HEM compounds [2]. Slipping and shifting of lattice planes normally occur when the crystal of the energetic compound is exposed to mechanical pressure. The resistance generated by the repulsion of positive charges between central regions of two HEM molecules induces the formation of hot spots—localized regions of thermal energy responsible for the detonation of energetic compounds. Another consequence of the existence of the strong positive potential in the central regions of the molecular surface is the withdrawal of electronic charge by the substituents attached to the aromatic ring of nitroaromatic energetic molecules. This withdrawal makes carbon-nitrogen bonds weaker, and the bond-breaking process occurs more easily. Practically, Klapötke demonstrated that electrostatic potential could be used to predict relative sensitivities towards detonation for energetic molecules with similar molecular frameworks [17,18,19,20].

Electrostatic potentials were also used to explain the results of the study of the detonation properties of energetic co-crystals, which indicate that TNT/CL-20 co-crystals have lower values of detonation velocity and reduced sensitivity towards detonation compared to isolated CL-20 molecules [21]. These differences were the consequence of the fact that positive potential in the central area of the CL-20 molecule decreases upon co-crystalisation of this molecule with TNT [21].

In our previous work, we demonstrated that the molecular electrostatic potential of square-planar acetylacetonato (abbreviation: M(acac)) chelate complexes of Ir, Rh, Pt, and Pd could be modified by careful selection of metal atoms and ligands [22]. Different combinations of ligands and metals in these complexes resulted in very different values of electrostatic potentials over the central regions of molecular surfaces. The obvious consequence of this was the existence of a very strong non-covalent O-H/M interaction between the metal atoms in these complexes and water molecules (−9.83 kcal/mol in case of O-H/Ir interaction between [Ir(acac)(en)]. complex and water). This procedure of combining metal atoms and ligands in order to adjust electrostatic potentials could be used for the development of the new energetic molecules based on the chelate coordination compounds.

Chelate complexes of Co, Zn, and Ni with π-stacking interactions and hydrogen bonds in their crystal structures were recently proposed as possible safer energetic materials [4]. Sensitivity tests and thermal studies were performed for three high energy coordination compounds: [Co(SCZ)_2_(H_2_O)_2_]·(TNR)(H_2_O)_2_, [Zn(SCZ)_2_(H_2_O)_2_]·(TNR)(H_2_O)_2_, and [Ni(SCZ)_2_(H2O)_2_].(TNR)(H_2_O)_2_ (where SCZ = semicarbazide and H_2_TNR = styphnic acid) and the results demonstrated that these energetic molecules are relatively insensitive towards mechanical stimuli [4]. It is especially important to point out the role of π-stacking interactions and hydrogen bonds for the control of the sensitivities of HEM compounds. In our recent study, it was found that hydrogen bonding can modify the values of positive electrostatic potential in the central areas of common HEM molecules (1,3,5-trinitrobenzene, 2,4,6-trinitrophenol, and 2,4,6-trinitrotoluene) up to 25% [23].

Another important example of the chelate energetic compounds represents the case of the Fe(II) complex with tetrazine/triazolo-tetrazine ligands and perchlorate as counter ions, which falls in the category of secondary explosives [5]. The study of the detonation characteristics of these complexes demonstrated that they are less sensitive than other secondary explosives, like pentaerythriol tetranitrate. An additional advantage of this class of chelate HEM molecules is the fact that they could be ignited using laser lights with lower energies compared to the laser lights used to ignite classical energetic molecules [5].

In this work, we used Density Functional Theory (DFT) calculations to analyze electrostatic potentials and Bond Dissociation Energies (BDE) for a series of nitro-bis(acetylacetonato) complexes in the context of their sensitivity towards detonation and possible application as energetic molecules. Since, in our previous work, we demonstrated that values of the electrostatic potential of chelate complexes could be easily tuned by combining different metal ions and ligands [22], this approach could be used as a new tool for the design of new classes of HEM compounds based on chelate complexes.

## 2. Results and Discussion

### 2.1. Molecular Electrostatic Potential Analysis

**Figure 1:**
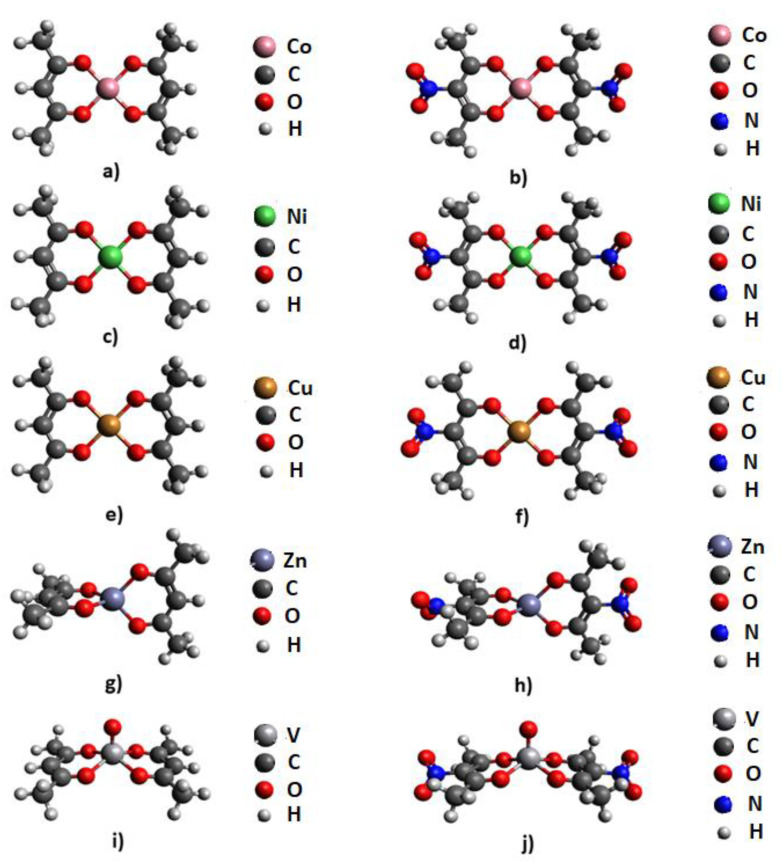
Optimized geometries for: (**a**) bis(acetylacetonato) cobalt (II), (**b**) nitro-bis(acetylacetonato) cobalt (II), (**c**) bis(acetylacetonato) nickel (II), (**d**) nitro-bis(acetylacetonato) nickel (II), (**e**) bis(acetylacetonato) copper (II), (**f**) nitro-bis(acetylacetonato) copper (II), (**g**) bis(acetylacetonato) zinc (II), (**h**) nitro-bis(acetylacetonato) zinc (II), (**i**) bis(acetylacetonato) oxovanadium (IV), and (**j**) nitro-bis(acetylacetonato) oxovanadium complexes.

Since it is known that values of positive potential in the middle regions of HEM molecules are a good indicator of their sensitivity towards detonation, Molecular Electrostatic Potentials (MEP) were calculated for five bis(acetylacetonato) and five nitro-bis(acetylacetonato) complexes. Geometries of these complexes were optimized prior to the MEP calculations and optimized structures are provided in Figure 1.

After geometry optimization, structures of bis(acetylacetonato) and nitro-bis(acetylacetonato) complexes of Co (II), Ni (II), and Cu (II) adopted square-planar geometry (Figure 1a–f), bis(acetylacetonato) and nitro-bis(acetylacetonato) complexes of zinc (II) adopted tetrahedral geometry (Figure 1g,h), while bis(acetylacetonato) and nitro-bis(acetylacetonato) oxovanadium (IV) complexes adopted square-pyramidal geometry (Figure 1i,j).

To compare calculated geometries with experimental data, we searched experimentally determined crystal structures of studied molecules in the Cambridge Structural Database. We found only crystal structures of bis(acetylacetonato) complexes, and we compared experimental geometries with our optimized geometries. We extracted and analyzed the following crystal structures (Figure S1): LIYLIO (bis(acetylacetonato) cobalt (II)), FEVMUP (bis(acetylacetonato) nickel (II)), ACACCU01 (bis(acetylacetonato) copper (II)), ASOCOC (bis(acetylacetonato) zinc (II)), and ACACVO07 (bis(acetylacetonato) oxovanadium (IV)). Analysis demonstrated that, in the crystal structures of the bis(acetylacetonato) complexes of Co, Ni, and Cu, geometries are square planar, which is in agreement with the geometries obtained by our calculations. The geometry of the bis(acetylacetonato) zinc (II) complex was tetrahedral and the geometry of the bis(acetylacetonato) oxovanadium (IV) complex was square-pyramidal in crystal structures, which is also in agreement with the results of our calculations.

Analysis of the calculated infrared spectra was performed using the Avogadro program (vibrations were visualized and animated for the purpose of the analysis). Calculated vibrational frequencies could be grouped into three regions: vibrations below 700 cm^−1^, from 700 cm^−1^ to 1700 cm^−1^, and above 1700 cm^−1^ (Figures S2–S11). Bands in the region below 700 cm^−1^ (especially between 700 and 500 cm^−1^) are the consequence of the M-O stretching vibrations (*v*(MO)). Low-intensity vibrations with the frequencies around 950 cm^−1^ correspond to the C-CH_3_ stretching vibrations (*v*(C-CH_3_)). Vibrations in the interval 1500–1400 cm^−1^ are due to the C-N stretching vibrations (*v*(C-N)). Vibrational bands around 1600 cm^−1^ are related to the stretching C-O vibrations (*v*(CO)) accompanied with C=C stretching vibrations (*v*(C=C)). Vibrations in the interval 1700–1600 cm^−1^ are the consequence of the N-O vibrations (*v*(N-O)). In all studied complexes, low-intensity frequencies around 3000 cm^−1^ are due to C-H stretching vibrations (*v*(C-H)).

**Figure 2:**
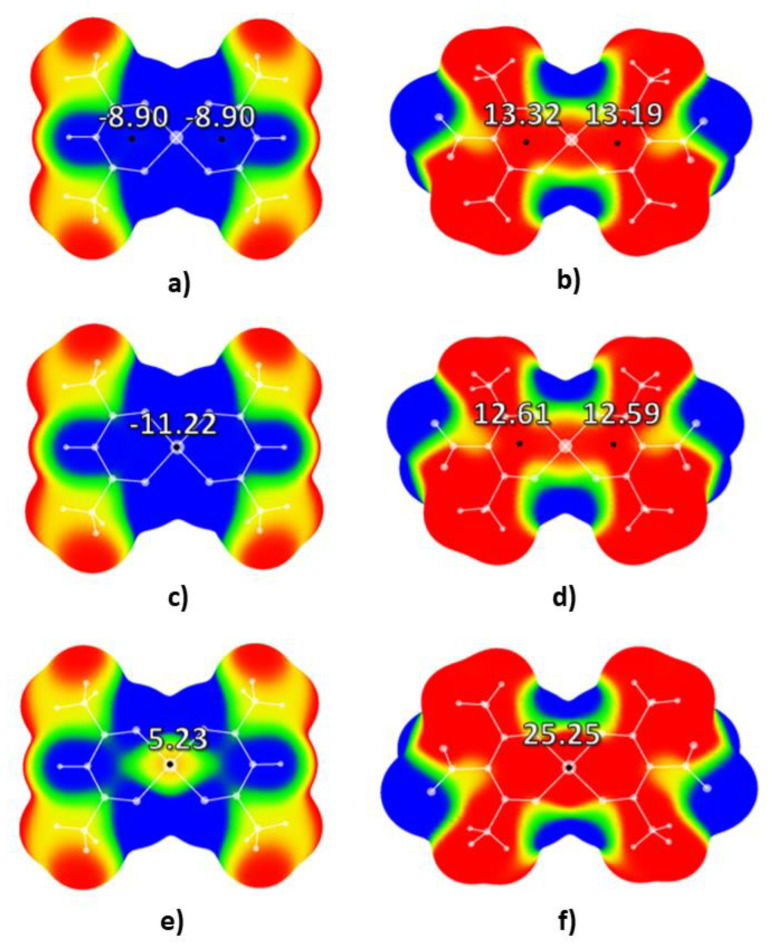
Calculated electrostatic potential maps for: (**a**) bis(acetylacetonato) cobalt (II), (**b**) nitro-bis(acetylacetonato) cobalt (II), (**c**) bis(acetylacetonato) nickel (II), (**d**) nitro-bis(acetylacetonato) nickel (II), (**e**) bis(acetylacetonato) copper (II), and (**f**) nitro-bis(acetylacetonato) copper (II) complexes. Values of energies in critical points are provided in kcal/mol. Color ranges, in kcal/mol, are: red, greater than 6.28, yellow, from 0.00 to 6.28, green, from −6.28 to 0.00, and blue, more negative than −6.28. Black dots represent critical points on the molecular surfaces.

Calculated electrostatic potential maps for square-planar bis(acetylacetonato) and nitro-bis(acetylacetonato) complexes are provided in Figure 2.

Analysis of the electrostatic potential values in the central regions of studied molecules demonstrated that positive potential in the centers of chelate molecules increases upon the addition of -NO_2_ groups to acetylacetonato ligands. Electrostatic potential in the central regions of bis(acetylacetonato) complexes of Co (II) and Ni (II) are negative (values of electrostatic potential in two critical points in the central region of bis(acetylacetonato) cobalt (II) complex are −8.90 kcal/mol each, while in one critical point in the center of bis(acetylacetonato) nickel (II) complex value of electrostatic potential is −11.22 kcal/mol). In the case of bis(acetylacetonato), the copper (II) complex value of electrostatic potential in the critical point located at the center of the molecule is slightly positive: 5.23 kcal/mol. Upon the addition of -NO_2_ substituents to the acetylacetonato ligands, electrostatic potential in the centers of all three bis(acetylacetonato) complexes, as presented in Figure 1, significantly increases. In the case of the nitro-bis(acetylacetonato) cobalt (II) complex, the values of electrostatic potential increase in both critical points to 13.32 and 13.19 kcal/mol. The most positive potential was calculated for the critical point in the center of the nitro-bis(acetylacetonato) copper (II) complex: 25.25 kcal/mol. This is more positive than the electrostatic potential previously calculated for the critical point in the center of the well-known conventional explosive 2,4,6-trinitotoluene (23.76 kcal/mol) [23]. This value is also very close to the values of electrostatic potentials in the central regions of other common explosives like 1,3,5-trinitrobenzene (27.33 kcal/mol) or 2,4,6-trinitrophenol (27.49 kcal/mol) [23].

It is also important to note that, for all three nitro-bis(acetylacetonato) complexes, positive (yellow) areas could be identified above the C-NO_2_ bonds. Positive potential above the bonds that are most likely to be broken during the detonation process is also a strong indicator of their sensitivity towards detonation.

**Figure 3:**
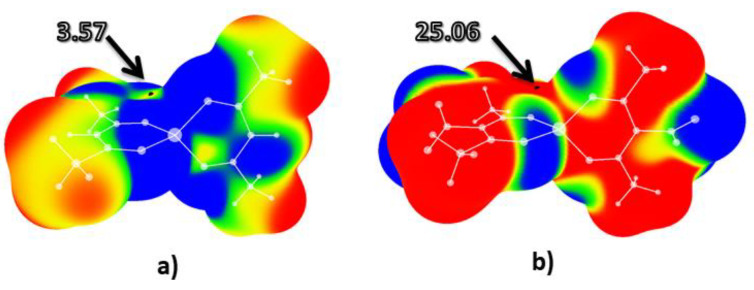
Calculated electrostatic potential maps for: (**a**) bis(acetylacetonato) zinc (II), and (**b**) nitro-bis(acetylacetonato) zinc (II) complexes. Color ranges, in kcal/mol, are: red, greater than 6.28, yellow, from 0.00 to 6.28, green, from −6.28 to 0.00, and blue, more negative than −6.28. Black dots represent critical points on the molecular surfaces.

Calculated electrostatic potential maps for tetrahedral bis(acetylacetonato) zinc (II) and nitro-bis(acetylacetonato) zinc (II) complexes are provided in Figure 3.

Results demonstrated that the value of electrostatic potential in the center of the bis(acetylacetonato) zinc (II) complex (Figure 3a) was slightly positive: 3.57 kcal/mol. In the case of the nitro-bis(acetylacetonato) zinc (II) complex (Figure 3b), positive potential increased to 25.06 kcal/mol. This value is more positive than the previously calculated value of electrostatic potential [23] in the center of 2,4,6-trinitotoluene (23.76 kcal/mol) and similar to the value calculated for the nitro-bis(acetylacetonato) copper (II) complex (25.25 kcal/mol). In the case of the nitro-bis(acetylacetonato) zinc (II) complex, a positive (yellow) area could be identified above the C-NO_2_ bond, similar to the other nitro-bis(acetylacetonato) complexes.

**Figure 4:**
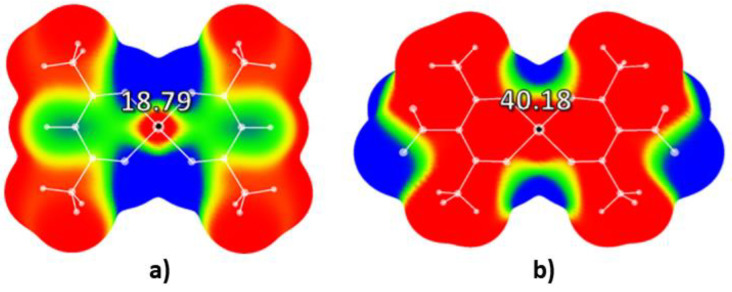
Calculated electrostatic potential maps for (**a**) bis(acetylacetonato) and (**b**) nitro-bis(acetylacetonato) oxovanadium (IV) complexes. Color ranges, in kcal/mol, are: red, greater than 6.28, yellow, from 0.00 to 6.28, green, from −6.28 to 0.00 kcal/mol, and blue, more negative than −6.28. Black dots represent critical points on the molecular surfaces.

Especially interesting are the cases of the bis(acetylacetonato) and nitro-bis(acetylacetonato) oxovanadium (IV) complexes. Calculated electrostatic potential for bis(acetylacetonato) oxovanadium (IV) complex (Figure 4a) shows relatively strong positive potential in the center of the molecule (18.79 kcal/mol). Upon the addition of the nitro-group to the acetylacetonato ligands, the value of electrostatic potential in the central region of nitro-bis(acetylacetonato) oxovanadium (IV) complex (Figure 4b) rises to 40.18 kcal/mol. This is significantly higher compared to all mentioned conventional explosives (2,4,6-trinitotoluene, 1,3,5-trinitrobenzene, and 2,4,6-trinitrophenol) [23]. In the case of the nitro-bis(acetylacetonato) oxovanadium (IV) complex there are also strongly positive (red) areas of positive electrostatic potential above the C-NO_2_ bonds.

Although -NO_2_ group is the most important group that affects the properties of nitro-energetic molecules, other substituents can affect the electrostatic potential of energetic molecules, too. It is known that the addition of -CH_3_ substituent to the aromatic ring of 1,3,5-trinitrobenzene leads to a decrease in the value of electrostatic potential in the central region of this molecule, from 27.33 to 23.76 kcal/mol [23]. It could be assumed that -CH_3_ groups attached to the chelate rings of studied complexes have a similar effect on the values of the electrostatic potential above the central area of the molecular surface. For the complexes with the strongest positive potentials in the central molecular regions (bis(acetylacetonato) and nitro-bis(acetylacetonato) oxovanadium (IV) complexes) we substituted -CH_3_ groups with H atoms, and we performed additional calculations of electrostatic potentials (Figure S12). Electrostatic potential above the center of modified the bis(acetylacetonato) oxovanadium (IV) complex (-CH_3_ groups substituted with H atoms, Figure S12a) was significantly more positive (29.95 kcal/mol) compared to the non-modified complex (18.79 kcal/mol, Figure 4a). Similar results were obtained for the modified nitro-bis(acetylacetonato) oxovanadium (IV) complex with the -CH_3_ group substituted with H atoms; the value of electrostatic potential above the central region of modified complex was 57.16 kcal/mol, which is significantly more positive than the electrostatic potential above the non-modified nitro-bis(acetylacetonato) oxovanadium (IV) complex (40.18 kcal/mol). Results of these calculations demonstrated that the -CH_3_ group strongly affects the value of the electrostatic potential of (bis(acetylacetonato) and nitro-bis(acetylacetonato) complexes.

However, since the positive value of electrostatic potential above the central portion of the molecular surface is only one of the indicators of the sensitivity towards detonation of potential HEM molecules, we calculated the bond dissociation energies (BDE) for C-NO_2_ groups of all studied nitro-bis(acetylacetonato) complexes. BDE values are a good indicator of how likely bond-breaking process is to occur. A combination of the results of the electrostatic potential analysis and the BDE calculation results can provide a good description of the detonation properties of energetic molecules.

### 2.2. Bond Dissociation Energies Calculations

Bond dissociation energies of C-NO_2_ bonds were calculated for all studied nitro-bis(acetylacetonato) complexes (abbreviation: M(acac-NO_2_)_2_). Calculated values of BDE with zero-potential energy correction are provided in Table 1. BDE values without zero-potential energy correction are provided in Table S11, in the Supplementary Materials section.

Analysis of the calculated bond dissociation energies demonstrates that the weakest C-NO_2_ bonds exist in the Zn(acac-NO_2_)_2_ complex: 58.09 kcal/mol. Similar BDE values were calculated for Co(acac-NO_2_)_2_, Ni(acac-NO_2_)_2_, and Cu(acac-NO_2_)_2_ complexes (58.99, 60.48, and 59.50 kcal/mol, respectively). In the case of VO(acac-NO_2_)_2_, complex BDE values for C-NO_2_ bonds were calculated to be higher: 78.17 kcal/mol. All calculated BDE values are similar to the BDE values of the classical explosives; for example, the calculated BDE value for the C-NO_2_ bond of 2,4,6-trinitrotoluene is 58.90 kcal/mol and for the C-NO_2_ bond of 1,3,5-triaminotrinitrobenzene is 69.40 kcal/mol [24]. The only exception is the nitro-bis(acetylacetonato) oxovanadium (IV) complex, in which the C-NO_2_ bond is somewhat stronger, indicating that the C-NO_2_ bond in this molecule is less likely to undergo the bond-breaking process. This is also in the contrast with the results of the analysis of electrostatic potential that indicate that VO(acac-NO_2_)_2_ complex is the most sensitive towards detonation since it has the most positive values of electrostatic potentials in the central regions of the molecular surface. It is important to note that higher values of BDE for the C-NO_2_ bond of VO(acac-NO_2_)_2_ complex are related to the lower sensitivity towards detonation, which would make VO(acac-NO_2_)_2_ a prospective candidate for the new energetic molecule with moderate sensitivity towards detonation.

## 3. Discussion

Results of the analysis of the electrostatic potentials for the series of bis(acetylacetonato) and nitro-bis(acetylacetonato) complexes of Ni, Co, Cu, Zn, and V demonstrated that the values of electrostatic potentials in the central areas of molecular surfaces become significantly more positive upon the addition of the NO_2_ groups to the acetylacetonato ligands. Results also demonstrated that fine-tuning of the electrostatic potential of chelate molecules could be achieved by changing the metal atom in the central parts of these complexes. Obtained results for studied nitro-bis(acetylacetonato) complexes demonstrated that nitro-bis(acetylacetonato) complexes of copper (II) and zinc (II) have electrostatic potential values in the central regions of molecules similar to the same values of conventional nitroaromatic explosives, while the nitro-bis(acetylacetonato) oxovanadium (IV) complex has significantly higher values of electrostatic potential in the center of the molecular surface. The strongest positive potential was calculated for the square-pyramidal VO(acac-NO_2_)_2_ complex (40.18 kcal/mol). The possible reason for this is the presence of the V=O fragment in the center of the molecule. The oxygen atom is capable of withdrawing negative charge, making the region in the extension of the V=O bond more positive than expected. This result indicates that M=O fragments in the central regions of square-pyramidal complexes may be used as a new tool for the adjustment of the values of the positive charge of the electrostatic potential of chelate energetic molecules. Results indicating that metal atoms may be used for the tuning of the electrostatic potential and the sensitivities of metal-containing energetic molecules are in agreement with the conclusions of previous studies related to the sensitivities of metal (metal = Mn, Co, Zn, and Cd) 1,5-diaminotetrazole perchlorate complexes [25].

Electrostatic potential maps were also analyzed in the regions above the C-NO_2_ bonds. In all studied complexes, electrostatic potential above the central region was positive, indicating that the bond-breaking process is likely to occur in the C-N regions. Electrostatic potentials above C-N bonds were yellow (positive) for all studied nitro-bis(acetylacetonato) complexes except for VO(acac-NO_2_)_2_, where the electrostatic potential map was red (strong positive charge). Since positive electrostatic potential in the central regions of the molecular surface and above C-N bonds is only one of the indicators of the sensitivity of high-energy molecules, bond dissociation energies for C-NO_2_ bonds of nitro-bis(acetylacetonato) complexes were also calculated and analyzed.

Analysis of the bond dissociation energies of C-NO_2_ bonds demonstrated that BDE values for studied complexes are similar to the BDE values of well-known nitroaromatic explosives like TNT. These results indicate that the nitro-bis(acetylacetonato) complexes of transition metals could be used as potential high-energy materials. This is consistent with the results of previous experimental studies demonstrating that nitro-substituted acetylacetonato complexes could act as energetic molecules [26,27]. Experimental study of the properties of the nitro-tris(acetylacetonato) aluminum (III) and nitro-tris(acetylacetonato) galium (III) complexes demonstrated that these compounds spontaneously ignite in air when heated [26]. Recently, authors found that the mixture of the nitroacetylacetonato complexes of In, Ga, and Zn could be used as a fuel for the combustion processing of indium-galium-zinc oxide thin films [27]. These molecules displayed higher enthalpies of combustion (988.6 vs. 784.4 J/g) and lower ignition temperatures (107.8 vs. 166.5 °C) in comparison with conventional acetylacetonato-based fuels [27].

Results obtained for the VO(acac-NO_2_)_2_ are conflicting and this complex needs to be studied in more detail in the future. While values of the electrostatic potentials above the central portion of the molecular surface and above the C-N bond indicate that this complex could be more sensitive towards detonation compared to other studied complexes, high values of BDE indicate that the bond-breaking process for the C-NO_2_ bond in this complex is less likely to occur compared to other studied complexes. It should be mentioned that the VO(acac-NO_2_)_2_ complexes have increased the amount of oxygen in the molecule due to the presence of the V=O fragment, which makes it an interesting candidate for the new type of energetic compounds, since oxygen content is also an important factor for the determination of the detonation properties of energetic molecules. Results of the calculations performed on modified structures of oxovanadium complexes demonstrated that the -CH_3_ group strongly affects the value of the electrostatic potential of bis(acetylacetonato) and nitro-bis(acetylacetonato) complexes.

## 4. Methodology

All geometry optimizations, wave function files calculations, and bond dissociation energies calculations were performed using Gaussian09 software [28]. To ensure that optimized geometries represent true minima, we calculated and analyzed vibrational spectra and determined that there are no imaginary frequencies. Coordinates of the optimized complexes (Tables S1–S10) and vibrational spectra (Figures S2–S11) are provided in the Supplementary Materials. Calculated wave function files were used to obtain electrostatic potential maps in the program WFA-SAS [29]. All geometry optimizations and wave function files calculations were performed using the M06 functional and cc-PVDZ basis set [30,31,32,33,34]. Bond dissociation energies calculations were performed using B3LYP functional and 6-311++G** according to the previously reported procedure [24]. The M06 method was used for geometry optimizations and electrostatic potential calculations, since our results were compared to the electrostatic potentials previously calculated with the same method [23]. The B3LYP method was used for bond dissociation energies calculations, since the BDE results of our calculations were compared to the BDE values of classical explosives previously calculated using, also, the B3LYP method [24]. Infrared spectra were corrected by the scaling factor of 0.975, which was previously used for the calculated IR spectra of metal acetylacetonates [35]. The visualization of three-dimensional structures of molecules and infrared spectra was performed using Avogadro and Mercury software [36]. The Cambridge Structural Database was searched to extract the crystal structures of acetylacetonato complexes [37].

## Figures and Tables

**Table 1:** Calculated bond dissociation energies (with zero-potential energy correction) of the weakest C-NO_2_ bonds in studied nitro-bis(acetylacetonato) complexes.

Complex	E _(complex)_^1^	E _(complex radical)_^1^	E _(NO2 radical)_^1^	BDE ^2^
Co(acac-NO_2_)_2_	−2482.339456	−2277.113539	−205.13191	58.99
Ni(acac-NO_2_)_2_	−2607.885082	−2402.656789	−205.13191	60.48
Cu(acac-NO_2_)_2_	−2740.057538	−2534.830809	−205.13191	59.50
Zn(acac-NO_2_)_2_	−2878.928600	−2673.704112	−205.13191	58.09
VO(acac-NO_2_)_2_	−2118.925282	−1913.668804	−205.13191	78.17

^1^ Energies provided in Hartrees. ^2^ Energies provided in kcal/mol.

## Supplementary Materials

The following are available online: Figure S1, Crystal structures extracted from the Cambridge Structural Database: LIYLIO (bis(acetylacetonato) cobalt (II)), FEVMUP (bis(acetylacetonato) nickel (II)), ACACCU01 (bis(acetylacetonato) copper (II)), ASOCOC (bis(acetylacetonato) zinc (II)), and ACACVO07 bis(acetylacetonato) oxovanadium (IV)). Figure S2, Calculated infrared spectrum for the optimized geometry of the Co(acac)2 complex. Figure S3, Calculated infrared spectrum for the optimized geometry of the Co(acac-NO2)2 complex. Figure S4, Calculated infrared spectrum for the optimized geometry of the Ni(acac)2 complex. Figure S5, Calculated infrared spectrum for the optimized geometry of the Ni(acac-NO2)2 complex. Figure S6, Calculated infrared spectrum for the optimized geometry of the Cu(acac)2 complex. Figure S7, Calculated infrared spectrum for optimized geometry of the Cu(acac-NO2)2 complex. Figure S8, Calculated infrared spectrum for the optimized geometry of the Zn (acac)2 complex. Figure S9, Calculated infrared spectrum for the optimized geometry of the Zn (acac-NO2)2 complex. Figure S10, Calculated infrared spectrum for the optimized geometry of the VO(acac)2 complex. Figure S11, Calculated infrared spectrum for the optimized geometry of the VO(acac-NO2)2 complex. Figure S12, Calculated electrostatic potential maps of modified bis(acetylacetonato) and nitro-bis(acetylacetonato) oxovanadium (IV) complexes (-CH3 groups substituted with H atoms). Table S1, Cartesian coordinates for the optimized geometry of the Co(acac)2 complex. Table S2: Cartesian coordinates for optimized geometry of the Co(acac-NO2)2 complex. Table S3, Cartesian coordinates for the optimized geometry of the Ni(acac)2 complex. Table S4, Cartesian coordinates for the optimized geometry of the Ni(acac-NO2)2 complex. Table S5, Cartesian coordinates for the optimized geometry of the Cu(acac)2 complex. Table S6, Cartesian coordinates for the optimized geometry of the Cu(acac-NO2)2 complex. Table S7, Cartesian coordinates for the optimized geometry of the Zn(acac)2 complex. Table S8, Cartesian coordinates for the optimized geometry of Zn(acac-NO2)2 complex. Table S9, Cartesian coordinates for the optimized geometry of VO(acac)2 complex. Table S10, Cartesian coordinates for the optimized geometry of VO(acac-NO2)2 complex. Table S11, Calculated bond dissociation energies (without zero-potential energy correction) of weakest C-NO2 bonds in studied nitro-bis(acetylacetonato) complexes.

## Sample Availability

Samples of compounds are not available from authors.

## References

[1] Thottempudi V., Gao H., Shreeve J.M. (2011). Trinitromethyl-Substituted 5-Nitro- or 3-Azo-1,2,4-triazoles: Synthesis, Characterization, and Energetic Properties. J. Am. Chem. Soc..

[2] Politzer P., Murray J.S. (2015). Some molecular/crystalline factors that affect the sensitivities of energetic materials: Molecular surface electrostatic potentials, lattice free space and maximum heat of detonation per unit volume. J. Mol. Model..

[3] Mathieu D. (2018). Atom Pair Contribution Method: Fast and General Procedure to Predict Molecular Formation Ethalpies. J. Chem. Inf. Model..

[4] Yang L., Tong W., Li H., Zhang G., Liu J. (2017). Chelates with π-stacking and hydrogen-bonding interactions as safer and structurally reinforced energetic materials. Inorg. Chim. Acta.

[5] Myers T.W., Bjorgaard J.A., Brown K.E., Chavez D.E., Hanson S.K., Scharff R.J., Tretiak S., Veauthier J.M. (2016). Energetic Chromophores: Low-Energy Laser Initiation in Explosive Fe(II) Tetrazine Complexes. J. Am. Chem. Soc..

[6] Joyner T.B. (1969). Explosive sensitivity of cobalt(III) ammine complexes. Can. J. Chem..

[7] Sinditskii V.P., Serushkin V.V. (2013). Design and Combustion Behaviour of Explosive Coordination Compounds. Def. Sci. J..

[8] Deblitz R., Hrib C.G., Blaurock S., Jones P.G., Plenikowski G., Edelmann F.T. (2014). Explosive Werner-type cobalt(III) complexes. Inorg. Chem. Front..

[9] Wojewódka A., Bełzowski J. (2011). Hydrazine complexes of transition metals as perspective explosives. Chemik.

[10] Young C.G., Volaric S. (2020). Synthesis, Iodometric Analysis, and IR Spectroscopy of the Peroxide Double Salt [Zn(NH_3_)_4_][Mo(O_2_)_4_]. J. Chem. Educ..

[11] Chhabra J.S., Talawar M.B., Makashir P.S., Asthana S.N., Singh H. (2003). Synthesis, characterization and thermal studies of (Ni/Co) metal salts of hydrazine: Potential initiatory compounds. J. Hazard. Mater..

[12] Liu G., Wei S.-H., Zhang C. (2020). Review of the Intermolecular Interactions in Energetic Molecular Cocrystals. Cryst. Growth Des..

[13] Politzer P., Murray J.S. (2016). High Performance, Low Sensitivity: Conflicting or Compatible?. Propellants Explos. Pyrotech..

[14] Politzer P., Murray J.S. (2014). Detonation Performance and Sensitivity: A Quest for Balance. Adv. Quantum Chem..

[15] Murray J.S., Lane P., Politzer P. (1998). Effects of strongly electron-attracting components on molecular surface electrostatic potentials: Application to predicting impact sensitivities of energetic molecules. Mol. Phys..

[16] Rice B.M., Hare J.J. (2002). A Quantum Mechanical Investigation of the Relation between Impact Sensitivity and the Charge Distribution in Energetic Molecules. J. Phys. Chem. A.

[17] Hammerl A., Klapötke T.M., Nöth H., Warchhold M. (2003). Synthesis, Structure, Molecular Orbital and Valence Bond Calculations for Tetrazole Azide. CHN_7_. Propellants Explos. Pyrotech..

[18] Hammerl A., Klapötke T.M., Mayer P., Weigand J.J., Holl G. (2005). Synthesis, Structure, Molecular Orbital Calculations and Decomposition Mechanism for Tetrazolylazide CHN7, its Phenyl Derivative PhCN7 and Tetrazolylpentazole CHN_9_. Propellants Explos. Pyrotech..

[19] Gökçınar E., Klapötke T.M., Bellamy A.J. (2010). Computational study on 2,6-diamino-3,5-dinitropyrazine and its 1-oxide and 1,4-dioxide derivatives. J. Mol. Struct. (THEOCHEM).

[20] Klapötke T.M., Nordheiter A., Stierstorfer J. (2012). Synthesis and reactivity of an unexpected highly sensitive 1-carboxymethyl-3-diazonio-5-nitrimino-1,2,4-triazole. New J. Chem..

[21] Li H., Shu Y., Gao S., Chen L., Ma Q., Ju X.J. (2013). Easy methods to study the smart energetic TNT/CL-20 co-crystal. J. Mol. Model..

[22] Janjić G.V., Milosavljevic M.D., Veljković D.Ž., Zarić S.D. (2017). Prediction of strong O–H/M hydrogen bonding between water and square-planar Ir and Rh complexes. Phys. Chem. Chem. Phys..

[23] Kretić D.S., Radovanović J.I., Veljković D.Ž. (2021). Can the sensitivity of energetic materials be tuned by using hydrgen bonds? Another look at the role of hydrogen bonding in the design of high energetic compounds. Phys. Chem. Chem. Phys..

[24] Rice B.M., Sahu S., Owens F.J. (2002). Density functional calculations of bond dissociation energies for NO_2_ scission in some nitroaromatic molecules. J. Mol. Struct. (THEOCHEM).

[25] Wang K., Zeng D., Zhang J., Cui Y., Zhang T., Jin X., Li Z. (2015). Controllable explosion: Fine-tuning the sensitivity of high-energy complexes. Dalton Trans..

[26] Djordjevic C. (1963). Aluminium(Ill) and Gallium(III) Tris-”f-Nitroacetylacetonates. Preparation and Infrared Spectra. Croat. Chem. Acta.

[27] Chen Y., Wang B., Huang W., Zhang X., Wang G., Leonardi M.J., Huang Y., Lu Z., Marks T.J., Facchetti A. (2018). Nitroacetylacetone as a Cofuel for the Combustion Synthesis of High-Performance Indium–Gallium–Zinc Oxide Transistors. Chem. Mater..

[28] Frisch M.J., Trucks G.W., Schlegel H.B., Scuseria G.E., Robb M.A., Cheeseman J.R., Scalmani G., Barone V., Petersson G.A., Nakatsuji H. (2016). Gaussian 09, Revision A.02.

[29] Bulat F.A., Toro-Labbe A., Brinck T., Murray J.S., Politzer P.J. (2010). Quantitative analysis of molecular surfaces: Areas, volumes, electrostatic potentials and average local ionization energies. Mol. Model..

[30] Zhao Y., Truhlar D.G. (2006). A new local density functional for main-group thermochemistry, transition metal bonding, thermochemical kinetics, and noncovalent interactions. J. Chem. Phys..

[31] Becke A.D. (1993). Density-functional thermochemistry. III. The role of exact exchange. J. Chem. Phys..

[32] Lee C., Yang W., Parr R.G. (1988). Development of the Colle-Salvetti correlation-energy formula into a functional of the electron density. Phys. Rev..

[33] Stephens P.J., Devlin F.J., Chabalowski C.F., Frisch M.J. (1994). Ab Initio Calculation of Vibrational Absorption and Circular Dichroism Spectra Using Density Functional Force Fields. J. Phys. Chem..

[34] Tsyshevsky R.V., Kuklja M.M. (2013). Decomposition mechanisms and kinetics of novel energetic molecules BNFF-1 and ANFF-1: Quantum-chemical modeling. Molecules.

[35] Houthuijs K.J., Martens J., Arranja A.G., Berden G., Nijsen J.F.W., Oomens J. (2020). Characterization of holmium(III)-acetylacetonate complexes derived from therapeutic microspheres by infrared ion spectroscopy. Phys. Chem. Chem. Phys..

[36] Hanwell M.D., Curtis D.E., Lonie D.C., Vandermeersch T., Zurek E., Hutchison G.R. (2012). Avogadro: An advanced semantic chemical editor, visualization, and analysis platform. J. Cheminformatics.

[37] Groom C.R., Bruno I.J., Lightfoot M.P., Ward S.C. (2016). The Cambridge Structural Database. Acta Crystallogr. Sect. B Struct. Sci. Cryst. Eng. Mater..

