# Supplementary material for: Chelate Coordination Compounds as a New Class of High-Energy Materials: The Case of Nitro-Bis(Acetylacetonato) Complexes
Source: Molecules. 2021 Sep 7;26(18):5438. doi: 10.3390/molecules26185438 (PMC8470082; doi:10.3390/molecules26185438)
Supplement: Supplementary file 1 [file molecules-26-05438-s001.zip › molecules-1359258-supplementary.pdf]

Supporting Information

Chelate Coordination Compounds as a New Class of High-Energey Materials. The Case of Nitro-Bis(Acetylacetonato) Complexes.

Danijela S. Kretić , Ivana S. Veljković, Aleksandra B. Đunović and Dušan Ž. Veljković *

I Crystal structures extracted from Cambridge Structural Database

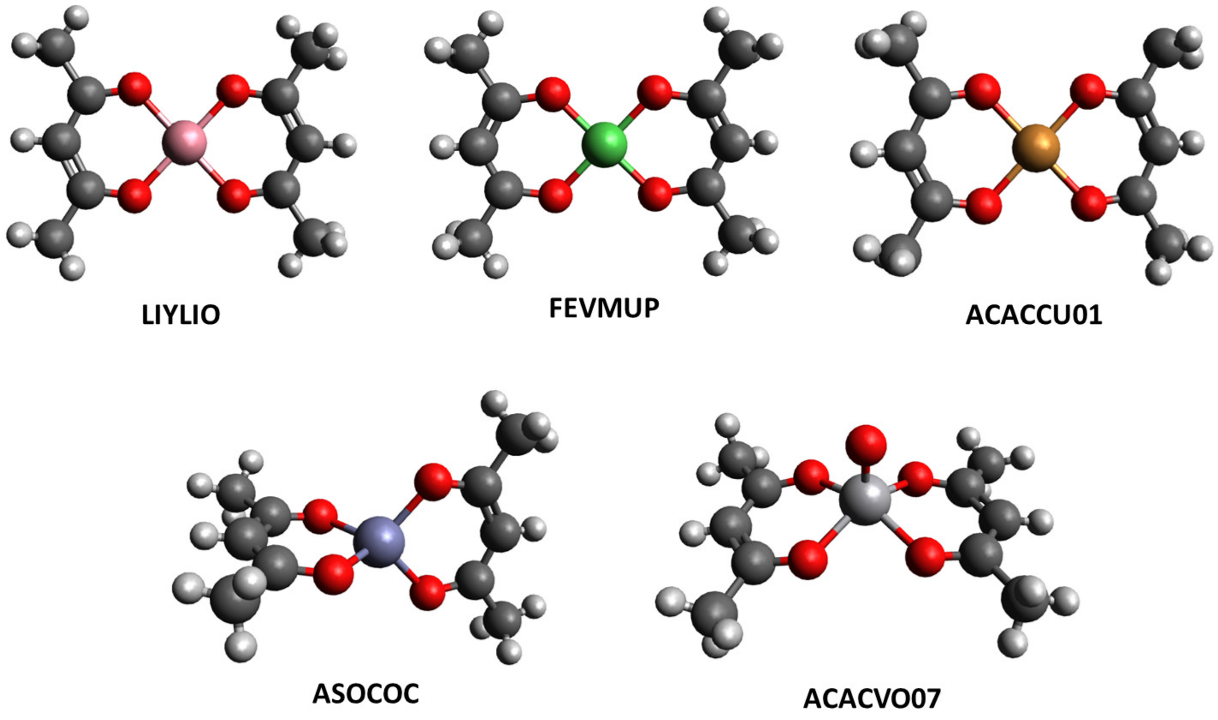

Figure S1. Crystal structures extracted from Cambridge Structural Database: LIYLIO (bis (acetylacetonato) cobalt (II)), FEVMUP (bis (acetylacetonato) nickel (II)), ACACCU01 (bis (acetylacetonato) copper (II)), ASOCOC (bis (acetylacetonato) zinc (II)) and ACACVO07 (bis (acetylacetonato) oxovanadium (IV)).

II Cartesian coordinates for optimized geometries of chelate complexes

Table S1. Cartesian coordinates for optimized geometry of $Co(acac)_2$ complex

atom	x	y	z
O	-1.27895	1.35443	0.00023
O	-1.27895	-1.35443	0.00018
C	-2.54669	1.22412	-0.00006
C	-3.22381	0.00000	-0.00021
C	-2.54669	-1.22412	-0.00003
C	-3.31200	2.50991	-0.00019
C	-3.31200	-2.50991	0.00005
H	-4.31461	0.00000	-0.00040
H	-3.02572	3.10294	-0.88267
H	-3.02575	3.10311	0.88218
H	-4.39885	2.35632	-0.00021
H	-4.39886	-2.35632	-0.00058
H	-3.02525	-3.10338	-0.88197
H	-3.02623	-3.10266	0.88288
H	3.02559	-3.10313	0.88227
H	3.02578	3.10297	0.88251
C	3.31200	-2.50991	-0.00003
H	4.39885	-2.35632	0.00016
C	2.54669	-1.22412	0.00001
C	3.22381	0.00000	0.00008
H	4.31461	0.00000	0.00015
O	1.27895	-1.35443	-0.00012
C	2.54669	1.22412	0.00000
C	3.31200	2.50991	0.00004
O	1.27895	1.35443	-0.00012
H	4.39886	2.35632	-0.00001
H	3.02588	-3.10292	-0.88257
H	3.02570	3.10308	-0.88233
Co	0.00000	0.00000	0.00005

Table S2. Cartesian coordinates for optimized geometry of $Co(acac\text{-}NO_2)_2$ complex

atom	x	y	z
O	-1.29948	1.34032	0.07189
O	-1.29310	-1.33806	-0.06695
C	-2.55875	1.25164	0.05448
C	-3.23500	-0.00255	0.00191
C	-2.56224	-1.24664	-0.04875
C	-3.27245	2.56241	0.13702
C	-3.26669	-2.56198	-0.12468
N	-4.69257	-0.00219	-0.00214
H	-3.64077	2.84411	-0.86033
H	-2.55508	3.31424	0.48768
H	-4.15368	2.52532	0.79008
H	-4.15996	-2.53257	-0.76106
H	-2.54847	-3.30725	-0.48753
H	-3.61378	-2.84917	0.87906
H	3.63974	-2.84510	0.85812
H	2.54843	3.30680	0.49030
C	3.27234	-2.56238	-0.13925
H	4.15419	-2.52472	-0.79149
C	2.55867	-1.25159	-0.05627
C	3.23495	0.00248	-0.00114
N	4.69255	0.00200	0.00509
O	1.29940	-1.34015	-0.07591
C	2.56230	1.24661	0.04946
C	3.26691	2.56175	0.12751
O	1.29313	1.33830	0.06569
H	4.15947	2.53158	0.76485
H	2.55530	-3.31377	-0.49152
H	3.61514	2.84988	-0.87557
Co	0.00000	0.00013	-0.00161
O	5.26023	-0.89511	0.60554
O	5.26871	0.89765	-0.5893
O	-5.26123	0.89438	-0.60255
O	-5.26777	-0.89735	0.59389

Table S3. Cartesian coordinates for optimized geometry of $Ni(acac)_2$ complex

atom	x	y	z
O	1.25847	-1.36345	0.00045
O	1.25850	1.36343	0.00030
C	2.52234	-1.22463	-0.00005
C	3.20021	-0.00002	-0.00039
C	2.52235	1.22463	-0.00001
C	3.29319	-2.50871	-0.00021
C	3.29326	2.50868	0.00018
H	4.29114	0.00000	-0.00068
H	3.00849	-3.10229	-0.88292
H	3.00874	-3.10235	0.88255
H	4.37954	-2.35029	-0.00038
H	4.37959	2.35020	-0.00086
H	3.00790	3.10302	-0.88179
H	3.00954	3.10159	0.88367
H	-3.00835	3.10254	0.88234
H	-3.00879	-3.10222	0.88293
C	-3.29321	2.50871	-0.00015
H	-4.37956	2.35028	0.00023
C	-2.52235	1.22463	-0.00011
C	-3.20021	0.00002	0.00012
H	-4.29115	-0.00002	0.00026
O	-1.25847	1.36345	-0.00028
C	-2.52235	-1.22463	0.00003
C	-3.29324	-2.50868	0.00011
O	-1.25849	-1.36343	-0.00013
H	-4.37958	-2.35021	-0.00004
H	-3.00895	3.10210	-0.88314
H	-3.00859	-3.10241	-0.88252
Ni	0.00000	0.00000	0.00002

Table S4. Cartesian coordinates for optimized geometry of Ni(acac-NO$_2$)$_2$ complex

atom	x	y	z
O	1.26654	1.33480	0.07266
O	1.26650	-1.33483	-0.07279
C	2.52887	1.24670	0.05095
C	3.20803	-0.00005	0.00000
C	2.52883	-1.24680	-0.05104
C	3.23084	2.56418	0.12684
C	3.23068	-2.56434	-0.12705
H	2.50668	3.31264	0.47051
H	3.60066	2.84109	-0.87120
H	4.11048	2.53684	0.78240
H	3.60049	-2.84137	0.87096
H	2.50641	-3.31270	-0.47074
H	4.11028	-2.53708	-0.78266
H	-2.50686	-3.31266	-0.47053
H	-3.60047	2.84125	-0.87115
H	-4.11066	-2.53674	-0.78219
C	-3.23092	-2.56414	-0.12675
N	-4.66362	-0.00002	-0.00007
C	-3.20799	0.00004	0.00000
C	-2.52887	-1.24671	-0.05099
C	-3.23078	2.56427	0.12692
C	-2.52883	1.24678	0.05106
O	-1.26654	-1.33481	-0.07269
H	-4.11049	2.53694	0.78238
O	-1.26650	1.33484	0.07279
H	-3.60062	-2.84102	0.87135
H	-2.50663	3.31271	0.47071
Ni	0.00000	0.00000	-0.00001
N	4.66367	0.00002	0.00009
O	5.23656	0.91211	-0.5728
O	5.23663	-0.91201	0.57298
O	-5.23649	-0.91233	0.57247
O	-5.23655	0.91231	-0.57254

Table S5. Cartesian coordinates for optimized geometry of $Cu(acac)_2$ complex

atom	x	y	z
O	-1.33117	-1.39535	0.00012
O	-1.33117	1.39535	-0.00013
C	-2.59111	-1.23407	0.00011
C	-3.25717	0.00000	0.00001
C	-2.59111	1.23407	-0.00011
C	-3.38994	-2.50336	0.00021
C	-3.38994	2.50336	-0.00036
H	-3.11455	-3.10228	0.88243
H	-3.11476	-3.10222	-0.88211
H	-4.47378	-2.32750	0.00032
H	-3.11439	3.10257	0.88161
H	-3.11492	3.10193	-0.88293
H	-4.47378	2.32750	0.00000
H	3.11495	3.10214	-0.88223
H	3.11459	-3.10207	-0.88274
H	4.47378	2.32750	0.00056
C	3.38994	2.50336	0.00020
C	3.25717	0.00000	-0.00006
C	2.59111	1.23407	0.00009
C	3.38994	-2.50336	-0.00037
C	2.59111	-1.23407	-0.00008
O	1.33117	1.39535	0.00026
H	4.47378	-2.32750	-0.00044
O	1.33117	-1.39535	-0.00002
H	3.11436	3.10236	0.88230
H	3.11472	-3.10242	0.88179
Cu	0.00000	-0.00000	0.00007
H	4.34848	0.00000	-0.00015
H	-4.34848	0.00000	0.00001

Table S6. Cartesian coordinates for optimized geometry of $Cu(acac\text{-}NO_2)_2$ complex

atom	x	y	z
O	-1.34631	-1.36085	0.06962
O	-1.34632	1.36084	-0.06928
C	-2.60569	-1.25496	0.05229
C	-3.27452	-0.00001	0.00004
C	-2.60569	1.25494	-0.05210
C	-3.33384	-2.56012	0.13508
C	-3.33385	2.56010	-0.13487
H	-2.62471	-3.31642	0.49312
H	-3.69508	-2.84205	-0.86483
H	-4.21981	-2.51544	0.78117
H	-3.69521	2.84196	0.86502
H	-2.62471	3.31643	-0.49280
H	-4.21977	2.51543	-0.78104
H	2.62437	3.31645	-0.49261
H	3.69529	-2.84202	-0.86508
H	4.21947	2.51572	-0.78124
C	3.33370	2.56020	-0.13488
N	4.73223	0.00001	0.00006
C	3.27457	-0.00001	-0.00002
C	2.60569	1.25496	-0.05201
C	3.33370	-2.56021	0.13474
C	2.60570	-1.25498	0.05187
O	1.34632	1.36082	-0.06937
H	4.21943	-2.51576	0.78118
O	1.34632	-1.36085	0.06913
H	3.69520	2.84205	0.86496
H	2.62436	-3.31650	0.49238
Cu	0.00000	-0.00001	0.00002
N	-4.73216	-0.00002	-0.00007
O	-5.30501	-0.88265	-0.61738
O	-5.30511	0.88273	0.617
O	5.30514	0.88203	0.61821
O	5.30523	-0.88193	-0.61813

Table S7. Cartesian coordinates for optimized geometry of $Zn(acac)_2$ complex

atom	x	y	z
H	-4.06654	2.37792	-1.13680
C	-3.41054	1.74784	-1.75790
H	-2.79019	2.38621	-2.39878
H	-4.07322	-1.11781	2.38002
C	-2.53967	0.88480	-0.89052
C	-3.17380	0.00266	-0.00015
H	-4.26757	0.00517	0.00052
O	-1.28443	1.02943	-1.03988
C	-2.54264	-0.88304	0.88877
C	-3.41636	-1.74373	1.75565
H	-4.06526	1.12321	-2.38586
O	-1.28789	-1.03356	1.03664
H	-2.79805	-2.38089	2.39970
H	-4.07022	-2.37531	1.13377
H	4.06891	-2.38378	-1.11791
C	3.41630	-1.75519	-1.74414
H	2.79799	-2.39557	-2.38497
H	4.06393	1.13796	2.37990
C	2.54264	-0.88871	-0.88302
C	3.17380	0.00258	0.00025
H	4.26758	0.00470	-0.00011
O	1.28786	-1.03981	-1.03016
C	2.53967	0.89074	0.88467
C	3.41059	1.75892	1.74688
H	4.07441	-1.13361	-2.37151
O	1.28446	1.03692	1.03261
H	2.79025	2.40235	2.38269
H	4.06796	2.38392	1.12213
Zn	0.00000	-0.00265	0.00002

Table S8. Cartesian coordinates for optimized geometry of Zn(acac-NO_2)$_2$ complex

atom	x	y	z
H	-4.24193	2.30994	-1.19750
C	-3.35018	1.91182	-1.69825
H	-2.66544	2.72240	-1.97666
H	-3.70448	-1.40818	2.60986
C	-2.58374	0.93937	-0.85710
C	-3.22783	-0.00008	0.00010
N	-4.69062	-0.00044	0.00015
O	-1.32545	1.04863	-0.94780
C	-2.58325	-0.93923	0.85727
C	-3.34919	-1.91193	1.69860
H	-3.70543	1.40798	-2.60948
O	-1.32491	-1.04796	0.94784
H	-2.66409	-2.72223	1.97694
H	-4.24089	-2.31040	1.19803
H	3.70367	-2.61074	-1.40733
C	3.34948	-1.69917	-1.91129
H	2.66478	-1.97699	-2.72212
H	4.24093	1.19772	2.31075
C	2.58340	-0.85738	-0.93911
C	3.22786	-0.00010	0.00003
N	4.69062	-0.00034	-0.00006
O	1.32509	-0.94768	-1.04829
C	2.58362	0.85755	0.93897
C	3.34985	1.69878	1.91154
H	4.24186	-1.19928	-2.30904
O	1.32531	0.94861	1.04769
H	2.66461	1.97810	2.72139
H	3.70606	2.60950	1.40746
Zn	0.00001	0.00051	-0.00019
O	5.2626	1.07092	0.11081
O	5.26214	-1.07187	-0.11077
O	-5.26233	-0.11186	1.0715
O	-5.26246	0.11051	-1.07119

Table S9. Cartesian coordinates for optimized geometry of $VO(acac)_2$ complex

atom	x	y	z
O	1.32541	1.35648	-0.08666
O	1.32597	-1.35643	-0.08618
C	3.25893	0.00040	-0.27239
C	2.58464	1.22712	-0.23744
C	2.58521	-1.22663	-0.23682
C	3.33940	2.50520	-0.41283
C	3.34050	-2.50447	-0.41157
H	3.16143	3.14828	0.46284
H	4.41796	2.35029	-0.54383
H	2.93493	3.04405	-1.28367
H	3.16125	-3.14806	0.46343
H	2.93759	-3.04292	-1.28340
H	4.41921	-2.34925	-0.54095
H	-4.41906	2.34941	-0.54002
C	-3.34020	2.50459	-0.41183
H	-3.15997	3.14894	0.46241
C	-2.58488	1.22678	-0.23680
C	-3.25875	-0.00021	-0.27160
O	-1.32561	1.35659	-0.08669
H	-3.15849	-3.14966	0.46090
H	-2.93811	3.04221	-1.28456
C	-2.58457	-1.22699	-0.23769
O	-1.32511	-1.35649	-0.08845
C	-3.33955	-2.50495	-0.41292
H	-2.93783	-3.04202	-1.28615
H	-4.41855	-2.35009	-0.54024
V	-0.00009	-0.00017	0.49699
H	-4.34021	-0.00030	-0.41310
H	4.34027	0.00062	-0.41475
O	-0.00099	-0.0005	2.06071

Table S10. Cartesian coordinates for optimized geometry of $VO(acac\text{-}NO_2)_2$ complex

atom	x	y	z
O	1.34218	1.32359	-0.06589
O	1.34058	-1.32611	0.08122
C	3.27582	-0.01505	-0.15423
C	2.60006	1.23395	-0.20124
C	2.59752	-1.26112	-0.07800
C	3.28987	2.52876	-0.47723
C	3.28693	-2.58166	-0.16731
H	3.68120	2.92957	0.47061
H	4.14743	2.43291	-1.15378
H	2.54412	3.22833	-0.87499
H	2.58304	-3.35397	0.16457
H	3.59358	-2.77342	-1.20587
H	4.20869	-2.60826	0.42876
H	-4.14702	2.43343	-1.15279
C	-3.29001	2.52905	-0.47552
H	-3.68201	2.92966	0.47213
C	-2.60048	1.23412	-0.19929
C	-3.27632	-0.01481	-0.15337
O	-1.34276	1.32373	-0.06253
H	-2.58505	-3.35330	0.17165
H	-2.54394	3.22871	-0.87251
C	-2.59840	-1.26090	-0.07463
O	-1.34173	-1.32602	0.08641
C	-3.28801	-2.58139	-0.16311
H	-3.59175	-2.77478	-1.20227
H	-4.21134	-2.60701	0.43049
V	0.00050	0.03243	0.60326
N	-4.72907	-0.00864	-0.27977
O	0.00364	0.11783	2.15880
O	-5.3358	0.90173	0.25774
O	-5.25931	-0.90857	-0.90862
N	4.72868	-0.00869	-0.27796
O	5.33419	0.9022	0.26015
O	5.26057	-0.90866	-0.90518

III Vibrational frequencies

Figure S2. Calculated infrared spectrum for optimized geometry of $Co(acac)_2$ complex

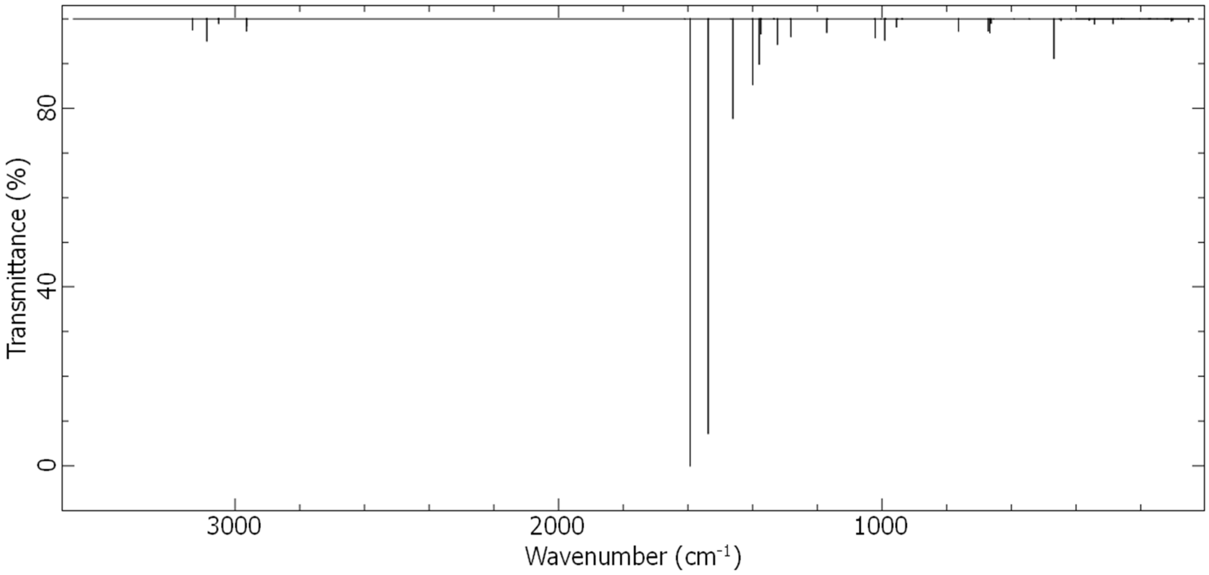

Figure S3. Calculated infrared spectrum for optimized geometry of $Co(acac\text{-}NO_2)_2$ complex

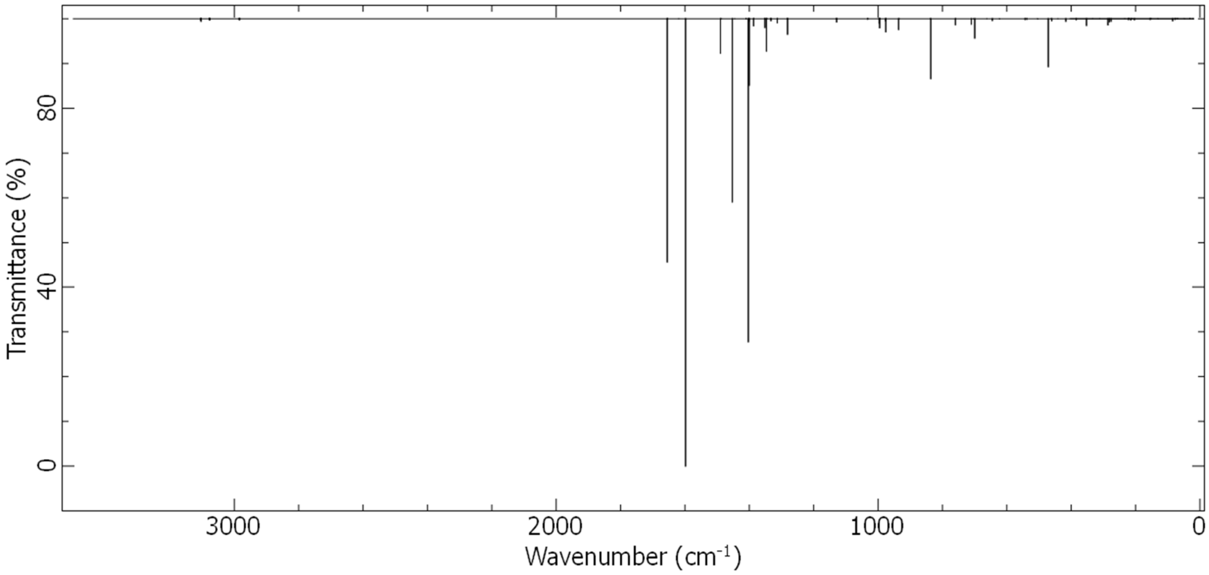

Figure S4. Calculated infrared spectrum for optimized geometry of Ni(acac)$_2$ complex

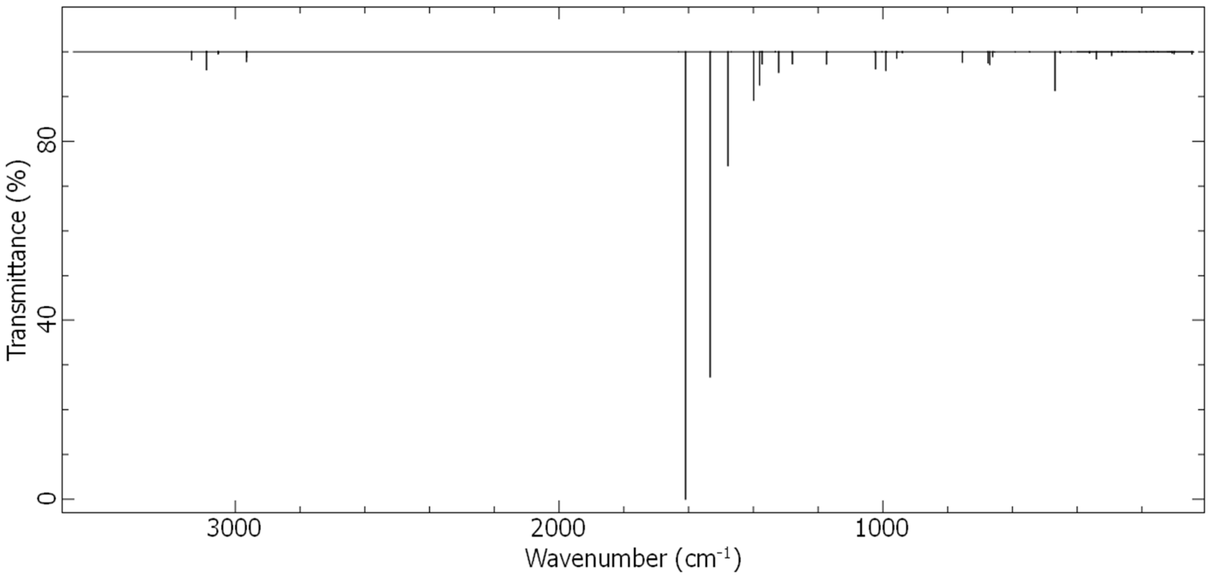

Figure S5. Calculated infrared spectrum for optimized geometry of Ni(acac-NO$_2$)$_2$ complex

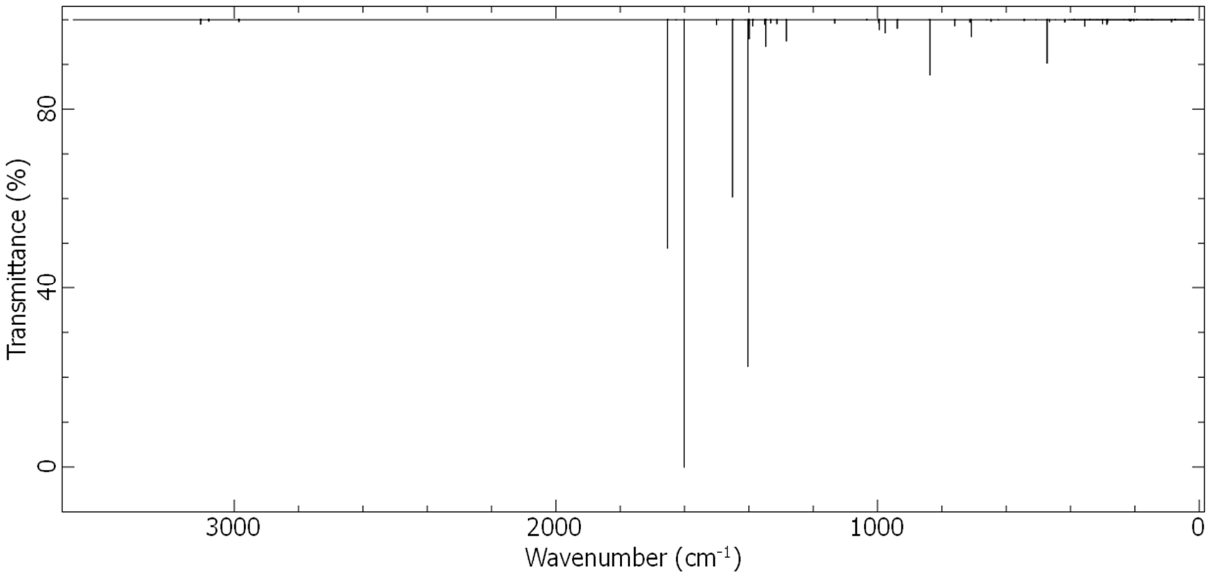

Figure S6. Calculated infrared spectrum for optimized geometry of Cu(acac)$_2$ complex

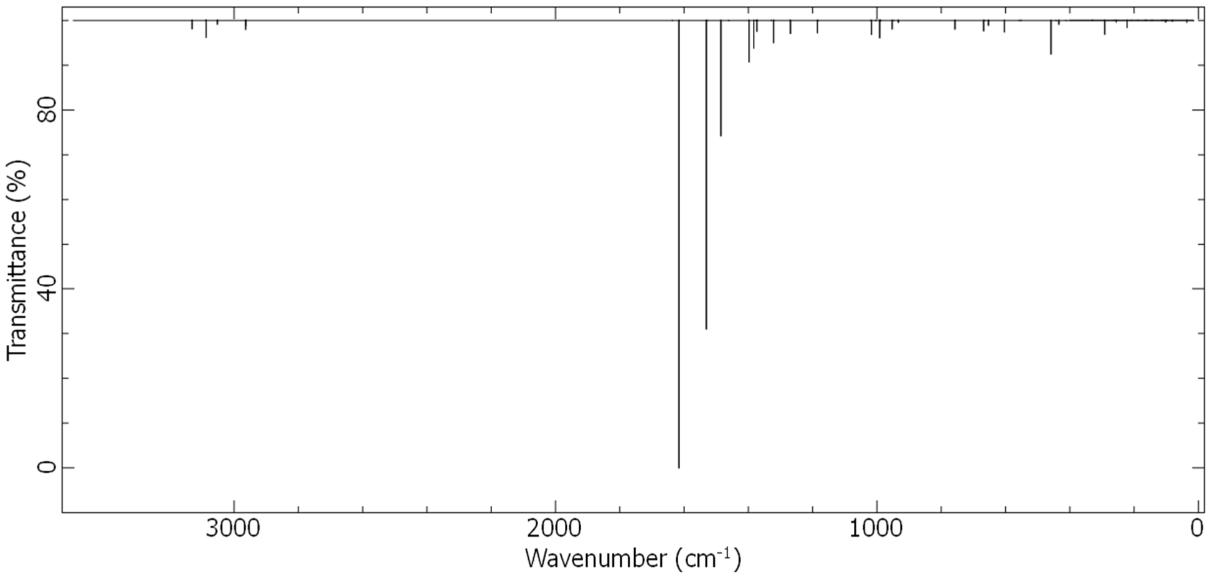

Figure S7. Calculated infrared spectrum for optimized geometry of Cu(acac-NO$_2$)$_2$ complex

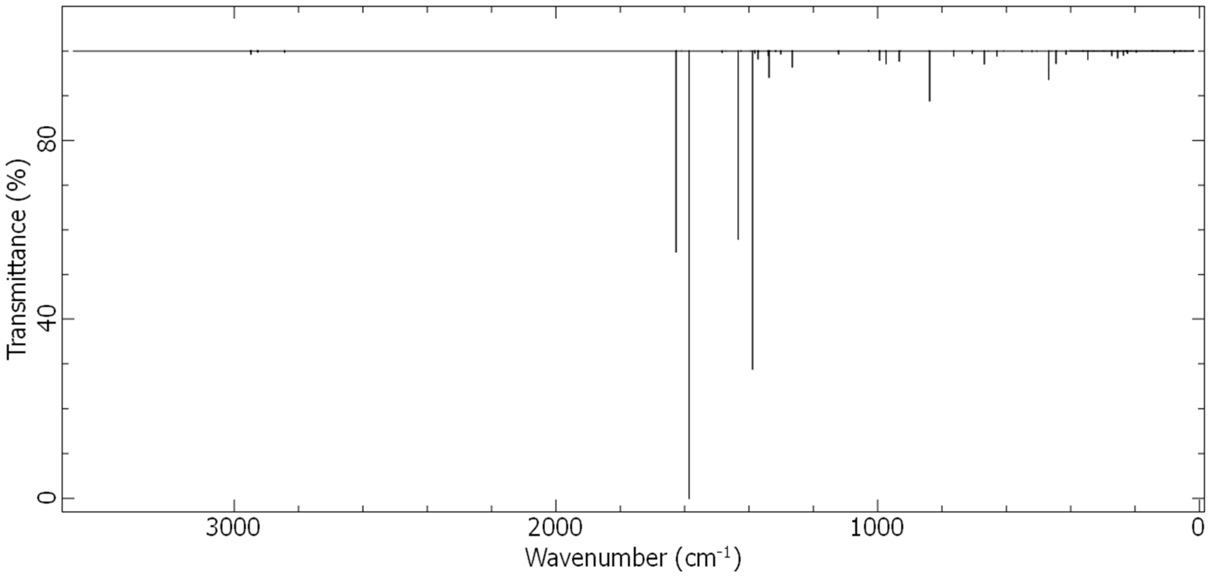

Figure S8. Calculated infrared spectrum for optimized geometry of $Zn(acac)_2$ complex

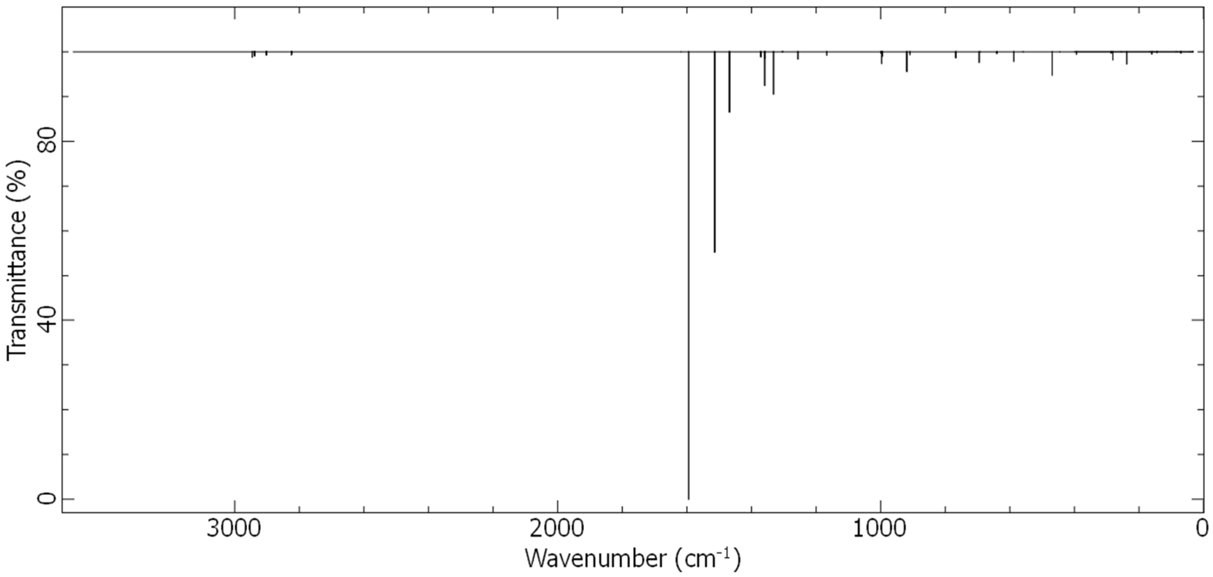

Figure S9. Calculated infrared spectrum for optimized geometry of $Zn(acac\text{-}NO_2)_2$ complex

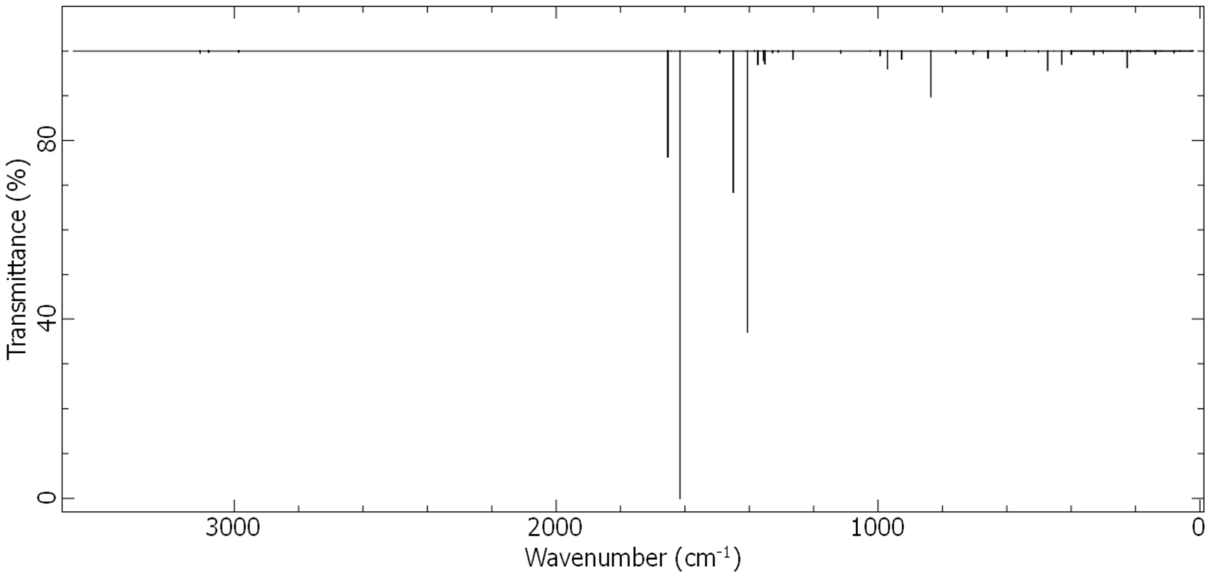

Figure S10. Calculated infrared spectrum for optimized geometry of $VO(acac)_2$ complex

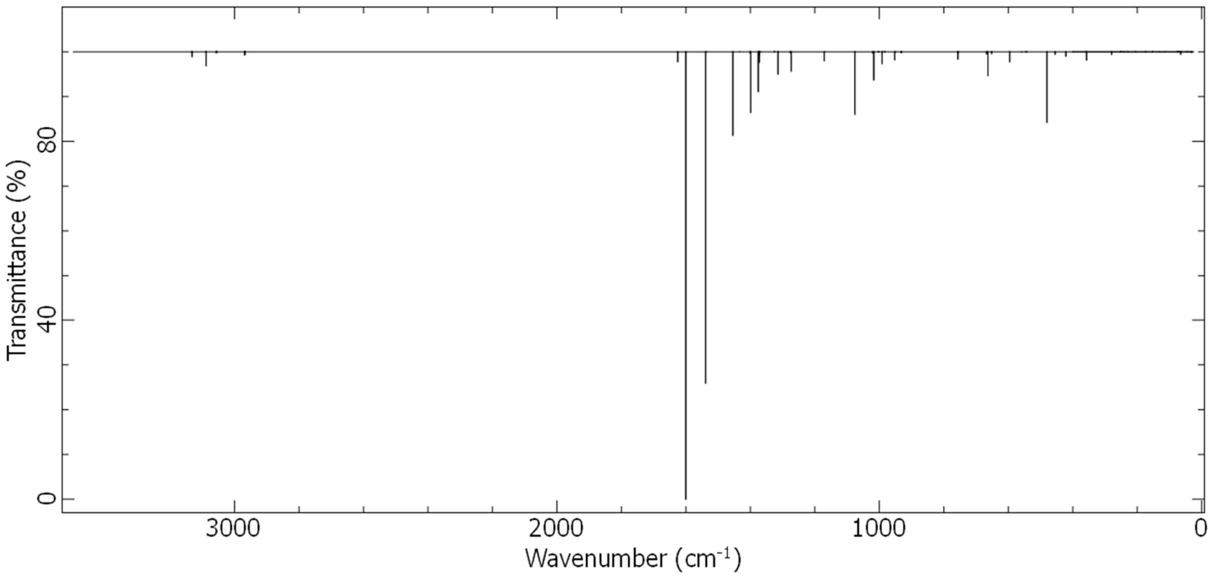

Figure S11. Calculated infrared spectrum for optimized geometry of $VO(acac\text{-}NO_2)_2$ complex

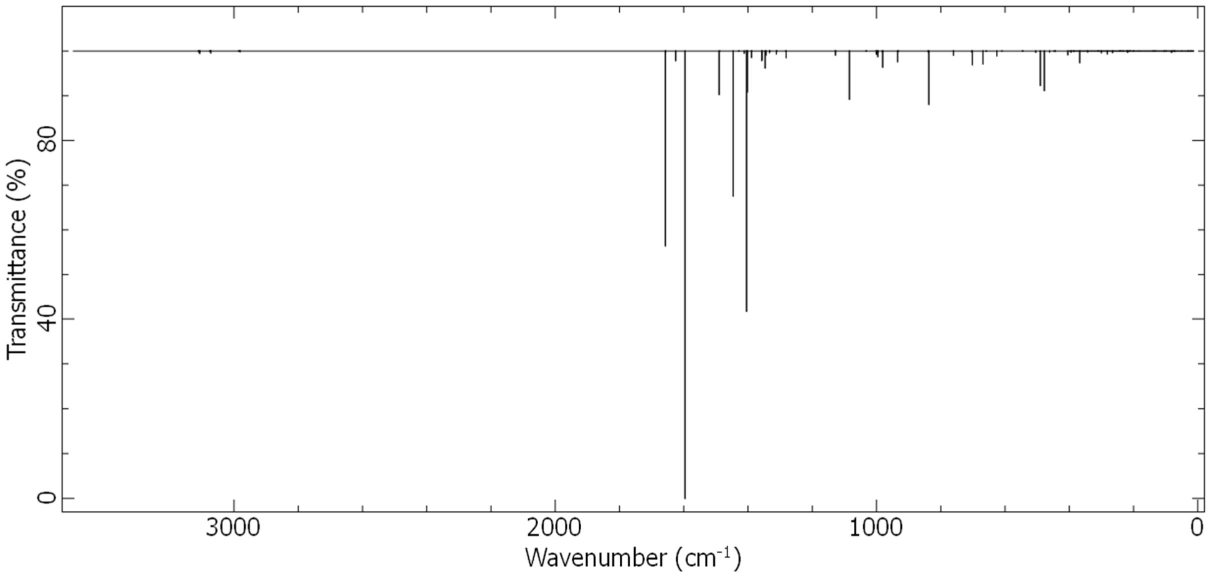

IV Bond dissociation energies (without zero-potential energy correction)

Table S11. Calculated bond dissociation energies (without zero-potential energy correction) of weakest C-NO_2 bonds in studied nitro-bis(acetylacetonato) complexes.

Complex	E (complex)[1]	E (complex radical)[1]	E (NO2 radical)[1]	BDE[2]
Co(acac-NO_2)$_2$	-2482.316510	-2277.092658	-205.128032	60.13
Ni(acac-NO_2)$_2$	-2607.862277	-2402.636087	-205.128032	61.60
Cu(acac-NO_2)$_2$	-2740.033947	-2534.809344	-205.128032	60.60
Zn(acac-NO_2)$_2$	-2878.904750	-2673.682471	-205.128032	59.14
VO(acacNO_2)$_2$	-2118.900795	-1913.646821	-205.128032	78.03

[1] Energies given in Hartrees
[2] Energies given in kcal/mol

V Calculated electrostatic potential maps for the modified structures of (bis (acetylacetonato) and nitro-bis (acetylacetonato) oxovanadium (IV) complexes

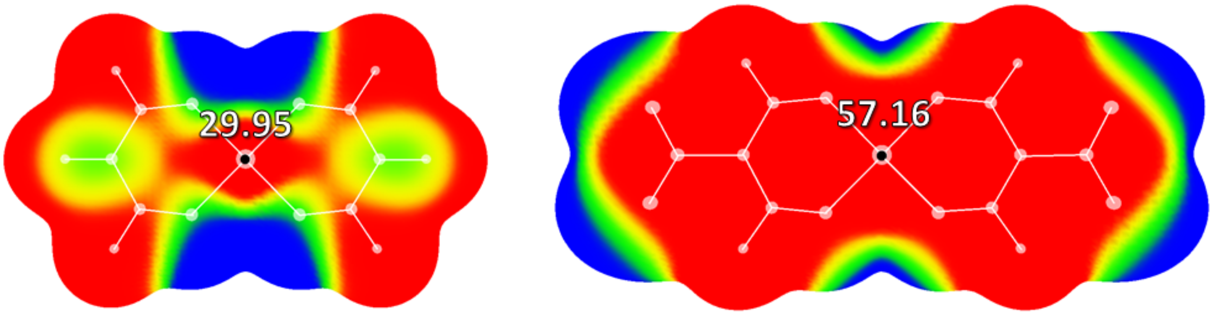

a) b)

Figure S12. Calculated electrostatic potential maps of modified bis (acetylacetonato) and nitro-bis (acetylacetonato) oxovanadium (IV) complexes (-CH_3 groups substituted with H atoms).
